# Sexual dysfunction in women with migraines: a systematic review

**DOI:** 10.1007/s00737-025-01650-6

**Published:** 2026-02-03

**Authors:** Karina Stech, Anupama Jayachandran, Huaqing Zhao, Behnum Habibi

**Affiliations:** 1 Lewis Katz School of Medicine at Temple University, Philadelphia, PA USA; 2 Department of Biomedical Education and Data Science, Lewis Katz School of Medicine at Temple University, Philadelphia, PA USA; 3 Department of Physical Medicine and Rehabilitation, Temple University Hospital, Philadelphia, PA USA

**Keywords:** Depression, Anxiety, Sexual Dysfunction, Sexual Intercourse, Quality of Life

## Abstract

**Purpose:**

The goal of this systematic review is to establish the prevalence of sexual dysfunction among women with migraines. Secondarily, this review explores factors contributing to the relationship between sexual dysfunction and migraine in women.

**Methods:**

A thorough search of PubMed from inception to June 17, 2024 was completed. Search terms included migraine related terms and sexual dysfunction outcome related terms. Two independent reviewers assessed articles for relevance and extracted information from selected articles.

**Results:**

In this review, a thorough search of the MEDLINE and CINAHL databases yielded 16 studies with 1,715 participants. The results demonstrated prevalence rates of sexual dysfunction in female migraine patients between 22.6% and 90%. There was a pooled prevalence of 69% (95% CI 61–77%) with substantial between-study heterogeneity (I^2^ = 78.73%). Prevalence rates of sexual dysfunction were higher in women with migraine than in healthy controls. Migraine characteristics, such as duration, frequency, and pain are associated with sexual dysfunction. A bi-directional correlation exists between psychological factors, such as anxiety and depression, and sexual dysfunction in patients with migraine.

**Conclusion:**

Migraines are associated with profound impacts on health-related quality of life. Sexual dysfunction is commonly associated with migraine in female patients, indicating that clinicians should routinely screen for sexual dysfunction in this population. Anxiety and depression may play a significant role in female sexual dysfunction in migraine.

**Supplementary Information:**

The online version contains supplementary material available at 10.1007/s00737-025-01650-6.

## Highlights

Sexual dysfunction is common in patients with migraines, and female sex is a major risk factor.

Migraine characteristics, like frequency and duration, may be associated with increased prevalence of sexual dysfunction.

Anxiety and depression may contribute to the relationship between migraines and sexual dysfunction.

There is no consensus on the relationship between headache-related pain and the degree of female sexual dysfunction.

**Supplementary Information:**

The online version contains supplementary material available at 10.1007/s00737-025-01650-6.

## Introduction

Migraine headaches are a prevalent neurologic condition that are more common in women, with a prevalence rate of 21% in women compared to 10.7% in men in the United States (Burch et al. 2021). Migraines are associated with profound impacts on health-related quality of life both during and between migraine episodes (Freitag 2007). Additionally, multiple comorbidities, including cardiovascular disease, psychiatric conditions including depression and anxiety, and sleep disorders, are associated with migraines, contributing to the significant disability associated with chronic migraine (Flegge et al. 2023).

Sexual function is an important component of quality of life and is often negatively impacted in the setting of chronic pain conditions (Flegge et al. 2023). Sexual dysfunction in both men and women is associated with poor physical and emotional health (Laumann et al. 1999; Lindau et al. 2007). Female sexual dysfunction (FSD) is common and entails a public health problem, highlighting the importance of further research on its causes (McCool et al. 2016).

Recent research indicates that the rate of sexual dysfunction is as high as 41.6% amongst patients with migraines (Torres-Ferrus et al. 2023). While a systematic review from 2022 on sexual dysfunction in migraine patients demonstrated increased rates of erectile dysfunction in male patients, a counterpart focusing on female patients has not yet been conducted (He et al. 2022). There are significant associations between female sex, chronic migraine status, and the development of sexual dysfunction. Female sex alone increased the risk of sexual dysfunction by more than double compared to male sex in the setting of migraine (Torres-Ferrus et al. 2023). The existing literature on sexual dysfunction in female migraine patients is primarily in the form of cross-sectional studies, often with very specific patient populations and limited size. There is a clear need for further study into the relationship between migraine and sexual dysfunction amongst female patients, yet there continues to be a gap in the literature to identify meaningful relationships that could guide clinical practice in managing migraine care.

This study evaluates the published literature relevant to the prevalence of sexual dysfunction amongst female patients with migraines and utilizes a meta-analysis approach to compare prevalence rates across studies. Additionally, it further explores factors that contribute to the development of sexual dysfunction in this population.

## Methods

### Systematic review protocol

The literature search methodology for this review was developed based on the Preferred Reporting Items of Systematic Reviews and Meta-Analyses (PRISMA) recommendations (Page et al. 2021). The review was not registered in PROSPERO because data extraction began prior to registration.

### Search strategy

The initial search identified studies in the MEDLINE and CINAHL databases through PubMed from inception to June 17, 2024 using a combination of keywords. Search terms included migraine related terms (e.g., “headache” and “migraine”) and outcome related terms including sexual dysfunction, sexual difficulty, sexual interest, sexual activity, sexual function, sexual relationship, and orgasm. The search used one migraine related term and one outcome related term with the Boolean operator “AND,” resulting in 14 different search combinations. A secondary search using the reference lists of included studies was conducted. The search was conducted with the aid of the Rayyan software which helped identify studies matching inclusion criteria and remove duplicate studies (Ouzzani et al. 2016). Searches were limited to those available in the English language. The studies were first screened by title and abstract to determine if inclusion criteria were met. The remaining studies were reviewed in full. Two reviewers (KS and AJ) independently assessed each study for eligibility. Any discrepancies in assessment were discussed before making a final unanimous decision. Cohen’s kappa score for overall screening was calculated as 0.6787, indicating substantial agreement between reviewers.

### Inclusion and exclusion criteria

Searches were limited to articles available in the English language. Case reports, descriptive studies, and review articles were excluded from the review and labeled as wrong study design. Eligible studies included data on sexual dysfunction in female migraine patients who were not undergoing interventions that could impact their FSD status. There were no age restrictions on the patient populations for this review.

Studies were excluded if they pooled migraine patients with other headache disorder patients or if they pooled male and female migraine patients. Studies relating to medication induced sexual dysfunction were also excluded. Non-human studies were excluded from this review.

### Quality assessment

The studies included in this review were assessed for bias in accordance with the Joanna Briggs Institute Critical Appraisal Checklist for Analytical Cross-Sectional Studies to evaluate their methodological quality (Aromataris et al. 2015; Moola et al. 2015). It was chosen because all the included studies were cross-sectional. Quality ratings were considered when assessing range of prevalence data.

### Data extraction

Data from the studies included were extracted into a pre-designed Excel spreadsheet. Data were collected from the methods, results, and discussion sections of the included studies. Studies were examined based on reported values on sexual dysfunction in comparison to control groups when available. Data on possible factors influencing the presence and severity of FSD were also extracted. Data extraction was completed independently by two reviewers (KS and AJ). Discrepancies were discussed between the two reviewers and revised accordingly.

### Statistical analysis

Pooling proportions were calculated as number of patients with migraine and sex dysfunction divided by the total number of patients. Freeman-Tukey double arcsine transformation and exact confidence intervals were employed for the individual studies. A random-effects model was applied to account for between-study variation.

## Results

### Search results

**Fig. 1:**
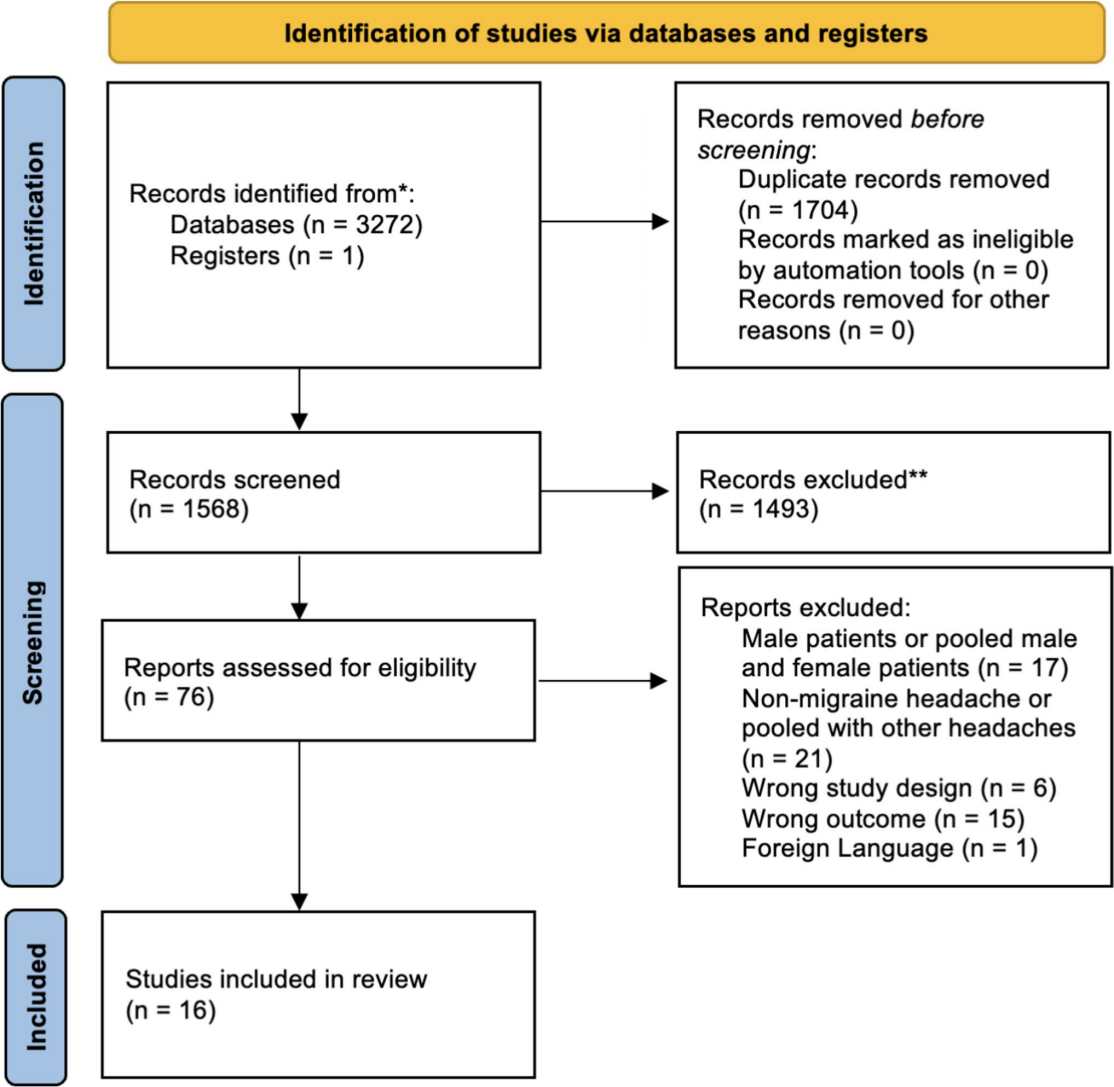
A PRISMA diagram depicting the search and retrieval process at different stages for this systematic review

The search yielded 3,272 articles. Seventy-six studies remained after removal of duplicates and the initial exclusion of studies based on the eligibility criteria. One additional study was added through screening the reference lists. A total of 16 studies met the eligibility criteria and were included in the review. The retrieval and search process is illustrated in Fig. 1.

### Study characteristics

Sixteen studies with a total of 1,715 participants were included in the review. The total number of participants in each study ranged from 50 to 180. All of the included studies consisted of exclusively female participants. The studies were published between 2008 and 2020 and were conducted in 7 countries including Turkey (*n* = 7), Iran (*n* = 3), India (*n* = 2), the United States (*n* = 1), Egypt (*n* = 1), Austria (*n* = 1), and Israel (*n* = 1). All sixteen studies were of cross-sectional design. Comparison groups included healthy participants (*n* = 9), tension type headache (TTH) (*n* = 5), and multiple sclerosis (*n* = 2). Of the five studies that used two comparison groups, all of them compared patients with migraines to healthy participants and participants with TTH. Characteristics of study design are detailed in Table 1.

**Table 1:** Characteristics of study design and sexual dysfunction assessment across all included studies

Study	Year	Country	Study design	Control group	Migraine patient sample size	Control sample size	Total sample size	FSD assessment tool
Abdollahi et al. (2015)	2015	Iran	Cross-sectional	N/A	57	-	57	FSFI
Ahmed et al. (2021)	2020	Egypt	Cross-sectional	- Tension Type Headache - Healthy	60	60 60	180	ArFSFI
Askari et al. (2016)	2016	Iran	Cross-sectional	Multiple Sclerosis	86	86	172	FSFI
Aydin et al. (2018)	2018	Turkey	Cross-sectional	- Tension Type Headache - Healthy	45	47 50	142	FSFI
Bestepe et al. (2011)	2011	Turkey	Cross-sectional	- Tension Type Headache - Healthy	44	30 30	104	ASEX
Bond et al. (2017)	2017	United States	Cross-sectional	Healthy	105	37	142	FSFI
Dogan et al. (2017)	2017	Turkey	Cross-sectional	Healthy	80	62	142	FSFI
Eraslan et al. (2014)	2014	Turkey	Cross-sectional	N/A	50	-	50	FSFI
Ertem et al. (2020)	2020	Turkey	Cross-sectional	Healthy	30	22	52	GRISS
Ghajarzadeh et al. (2014)	2014	Iran	Cross-sectional	N/A	100	-	100	FSFI
Ifergane et al. (2008)	2008	Israel	Cross-sectional	Healthy	32	95	127	ISBI
Kucukdurmaz et al. (2018)	2018	Turkey	Cross-sectional	N/A	69	-	69	FSFI
Nagpal et al. (2018)	2018	India	Cross-sectional	- Tension Type Headache - Healthy	50	45 30	125	FSFI
Pradeep et al. (2019)	2019	India	Cross-sectional	N/A	60	-	60	FSFI
Salhofer-Polanyi et al. (2017)	2016	Austria	Cross-sectional	Multiple Sclerosis	30	42	72	MSISQ-19
Solmaz et al. (2016)	2016	Turkey	Cross-sectional	- Tension Type Headache - Healthy	41	39 41	121	FSFI

*FSFI* Female Sexual Function Index

*ArFSFI* Arabic translation of FSFI

*ASEX* Arizona Sexual Experiences Scale

*GRISS* Golombok Rust Inventory of Sexual Satisfaction

*ISBI* Israeli Sexual Behavior Inventory

*MSISQ-19* Multiple Sclerosis Intimacy and Sexuality Questionnaire

### Assessment of sexual functioning

Assessment of sexual function in all studies relied on self-report tools. The Female Sexual Function Index (FSFI), a 19-item questionnaire that measures desire, subjective arousal, lubrication, orgasm, satisfaction, and pain, was the most used tool (*n* = 12) (Rosen et al. 2000). One study used the Arizona Sexual Experience Scale (ASEX), a 5-item scale that measures sex drive, arousal, vaginal lubrication, ability to reach orgasm, and satisfaction from orgasm (McGahuey et al. 2000). One study used the Golombok-Rust Inventory of Sexual Satisfaction (GRISS), a 28-item questionnaire that measures impotence, premature ejaculation, female anorgasmia, vaginismus, infrequency of sexual contact, sexual non-communication, dissatisfaction, non-sensuality, and avoidance of sex (Rust and Golombok 1985). One study used the Israeli Sexual Behavior Inventory (ISBI), a 41-item questionnaire that measures sexual satisfaction, intimacy, sexual drive, anorgasmia/avoidance, and vaginismus/dyspareunia (Kravetz et al. 1999). Finally, one study used the Multiple Sclerosis Intimacy and Sexuality Questionnaire (MSISQ-19), a 19-item questionnaire that measures marital satisfaction, neurological impairment and level of disability in multiple sclerosis, psychological distress and well-being, and global sexual dysfunction (Sanders et al. 2000).

### Assessment of other outcomes

Additional outcomes were measured in 14 studies to describe possible causes of change in sexual function in patients with migraines. Migraine characteristics and impact were measured in ten studies using the Migraine Disability Assessment Test (MIDAS; *n* = 6), Headache Impact Test (HIT; *n* = 2), headache duration (*n* = 1), and general headache characteristics assessment (*n* = 1). Pain was measured in nine studies using the Visual Analogue Scale (VAS; *n* = 7) and the Numeric Rating Scale (*n* = 2). Anxiety was measured in six studies using the Beck Anxiety Inventory (BAI; *n* = 3), the Generalized Anxiety Disorder Scale (GAD-7; *n* = 1), and the Hospital Depression and Anxiety Scale (HDAS; *n* = 2). Depression was measured in nine studies using the Beck Depression Inventory (BDI; *n* = 6), the Center for Epidemiologic Studies Depression Scale (CES-D; *n* = 1), and the Hospital Depression and Anxiety Scale (HDAS; *n* = 2). Hormone levels, including progesterone, prolactin, follicle stimulating hormone (FSH), and luteinizing hormone (LH) were measured in one study. Sociodemographic characteristics were measured in one study. Sleep quality was measured in one study using the Pittsburgh Sleep Questionnaire (PSQ). Details on assessment tools, sexual function outcomes, and secondary outcomes relating to sexual dysfunction are included in summary Table 2.

**Table 2:** Brief summary table of sexual dysfunction prevalence and secondary contributing factors

Study	Year	Country	Sexual function prevalence in migraine patients	Secondary outcomes contributing to sexual dysfunction
Abdollahi et al. (2015)	2015	Iran	68.4%	Headache frequency positively correlated with sexual dysfunction
Ahmed et al. (2021)	2020	Egypt	N/A	Headache pain, depression and anxiety positively correlated with sexual dysfunction
Askari et al. (2016)	2016	Iran	67%	Depression positively correlated with sexual dysfunction
Aydin et al. (2018)	2018	Turkey	65%	N/A
Bestepe et al. (2011)	2011	Turkey	N/A	No relationship between migraine characteristics and sexual dysfunction
Bond et al. (2017)	2017	United States	56.8%	Anxiety positively correlated with sexual disability
Dogan et al. (2017)	2017	Turkey	76.1%	Depression positively correlated with sexual dysfunction
Eraslan et al. (2014)	2014	Turkey	90%	No correlation between headache pain, migraine characteristics, anxiety and sexual dysfunction Depression positively correlated with sexual dysfunction
Ertem et al. (2020)	2020	Turkey	N/A	Migraine pain positively correlated with sexual dysfunction
Ghajarzadeh et al. (2014)	2014	Iran	68%	Depression associated with lower scores on components of sexual dysfunction
Ifergane et al. (2008)	2008	Israel	N/A	N/A
Kucukdurmaz et al. (2018)	2018	Turkey	79.7%	Headache pain, anxiety, and depression positively correlated with sexual distress
Nagpal et al. (2018)	2018	India	66.3%	Headache pain, headache characteristics, and depression positively correlated with sexual dysfunction
Pradeep et al. (2019)	2019	India	78.3%	Headache duration and frequency positively correlated with sexual dysfunction
Salhofer-Polanyi et al. (2017)	2016	Austria	22.6%	Depression positively correlated with sexual dysfunction
Solmaz et al. (2016)	2016	Turkey	75.6%	Migraine frequency positively correlated with sexual dysfunction

### Overall sexual function outcomes

All studies demonstrated some degree of sexual dysfunction among participants with migraines (see Table 2). Twelve studies discussed prevalence of sexual dysfunction in women with migraines (Abdollahi et al. 2015; Askari et al. 2016; Aydin et al. 2018; Bond et al. 2017; Dogan et al. 2017; Eraslan et al. 2014; Ghajarzadeh et al. 2014; Kucukdurmaz et al. 2018; Nagpal et al. 2018; Pradeep et al. 2019; Salhofer-Polyani et al. 2017; Solmaz et al. 2016). Prevalence rates ranged from 22.6% (Salhofer-Polyani et al. 2017) to 90% (Eraslan et al. 2014). Of the nine studies with healthy participant controls (Ahmed et al. 2021; Aydin et al. 2018; Bestepe et al. 2011; Bond et al. 2017; Dogan et al. 2017; Ertem et al. 2020; Ifergane et al. 2008; Nagpal et al. 2018; Solmaz et al. 2016), four demonstrated significantly greater prevalence rates of overall sexual dysfunction in patients with migraines compared to healthy controls (Aydin et al. 2018; Dogan et al. 2017; Nagpal et al. 2018; Solmaz et al. 2016) and six demonstrated significantly poorer average scores on measures of sexual dysfunction in patients with migraines (Ahmed et al. 2021; Bestepe et al. 2011; Dogan et al. 2017; Ifergane et al. 2008; Nagpal et al. 2018; Solmaz et al. 2016). Five studies demonstrated poorer scores in all domains of sexual function in patients with migraines (Ahmed et al. 2021; Aydin et al. 2018; Dogan et al. 2017; Nagpal et al. 2018; Solmaz et al. 2016). Two studies found no significant difference in prevalence of sexual dysfunction between migraine patients and healthy controls (Bond et al. 2017; Ertem et al. 2020).

### Migraine characteristics and sexual dysfunction

Of the ten studies that measured migraine characteristics and impact, six studies demonstrated a relationship between migraine characteristics and sexual dysfunction or distress. Total MIDAS/HIT-6 score was associated with sexual dysfunction (*n* = 2) (Ahmed et al. 2021; Nagpal et al. 2018), sexual distress (*n* = 1) (Kucukdurmaz et al. 2018), and decreased satisfaction (*n* = 1) (Dogan et al. 2017). One study demonstrated a decrease in the health domain of the sexual dysfunction measurement tool but not the total score (Ifergane et al. 2008). Three studies demonstrated a positive relationship between migraine frequency and sexual dysfunction (Abdollahi et al. 2015; Nagpal et al. 2018; Solmaz et al. 2016) and one study demonstrated decreased sexual satisfaction related to headache frequency (Dogan et al. 2017). One study measured migraine frequency in increments of weekly, once a week, and monthly and demonstrated an increased number of cases of sexual dysfunction as migraine frequency increased (Abdollahi et al. 2015). Additionally, duration of acute migraine episodes was associated with overall sexual dysfunction (*n* = 2) (Nagpal et al. 2018; Pradeep et al. 2019) and decreased desire (*n* = 1) (Pradeep et al. 2019). Of the nine studies that discussed pain and sexual dysfunction, three demonstrated that pain was associated with increased sexual dysfunction (Ahmed et al. 2021; Ertem et al. 2020; Nagpal et al. 2018) and one demonstrated increased sexual distress (Kucukdurmaz et al. 2018).

### Depression and sexual dysfunction

Of the nine studies that measured depression, seven studies demonstrated a positive correlation between depression and sexual dysfunction (Ahmed et al. 2021; Askari et al. 2016; Dogan et al. 2017; Eraslan et al. 2014; Ertem et al. 2020; Ghajarzadeh et al. 2014; Salhofer-Polyani et al. 2017). Of these, four studies utilized both FSFI and BDI to assess for sexual dysfunction and depression respectively in migraine patients. Correlation coefficients between these two outcomes are listed in Table 3, demonstrating an overall correlation between presence of depression and sexual dysfunction. Additional studies which utilized a different tool for recording depression noted a similar association with sexual dysfunction scores, mirrored in the association of depression with specific sub sects of the FSFI tool (Ahmed et al. 2021). Two studies additionally demonstrated associations between the development of depression and both migraine and sexual dysfunction (Ahmed et al. 2021; Askari et al. 2016). One study also demonstrated a depression prevalence rate of 85.7% amongst women with migraine and sexual dysfunction compared to a rate of 39.1% in women with migraine but without sexual dysfunction (Salhofer-Polyani et al. 2017).

**Table 3:** Pearson correlation values for BDI score to FSFI score in migraine patients that were reported as having a p value less than 0.05; x indicates non significant correlation coefficient

	FSFI score and subsets					
Study	Total score	Desire	Arousal	Lubrication	Orgasm	Satisfaction
Eraslan et al. (2014)	- 0.486	- 0.406	- 0.51	- 0.454	- 0.47	- 0.558
Ghajarzadeh et al. (2014)	- 0.39	- 0.33	- 0.25	- 0.27	- 0.24	- 0.25
Askari et al. (2016)	- 0.37	- 0.36	- 0.3	- 0.31	- 0.3	- 0.29
Dogan et al. (2017)	- 0.280	x	- 0.274	- 0.419	- 0.264	x

### Anxiety and sexual dysfunction

Of the six studies that measured anxiety, four demonstrated a relationship between anxiety and sexual dysfunction (Ahmed et al. 2021; Bond et al. 2017; Dogan et al. 2017; Ertem et al. 2020). One study demonstrated a 15% increase in the odds of sexual dysfunction among women with migraine and anxiety (Bond et al. 2017). Anxiety was associated with decreased arousal and lubrication in one study (Dogan et al. 2017) and associated with decreased satisfaction and increased avoidance, nonsensuality, anorgasmia, and vaginismus in another (Ertem et al. 2020). Additionally, one study demonstrated increased anxiety scores in female migraine patients with sexual dysfunction (Ahmed et al. 2021).

### Other factors impacting sexual dysfunction

One study explored the relationship between sleep quality, as measured by the PSQI, and sexual dysfunction (Ghajarzadeh et al. 2014). 79% of migraine patients in this study had poor sleep which was associated with depression. Sexual dysfunction increased the odds of developing poor sleep, but poor sleep had no impact on sexual dysfunction.

Another study explored the relationship between hormones (FSH, LH, progesterone, estrogen, and prolactin) and sexual dysfunction in migraine (Dogan et al. 2017). Progesterone levels were lower in women with migraine than healthy controls (*p* = 0.004) but were not associated with sexual dysfunction. Prolactin was lower in women with migraine than healthy controls (*p* = 0.016) and was positively correlated with desire (*p* = 0.023) and lubrication (*p* = 0.057).

### Statistical analysis

**Fig. 2:**
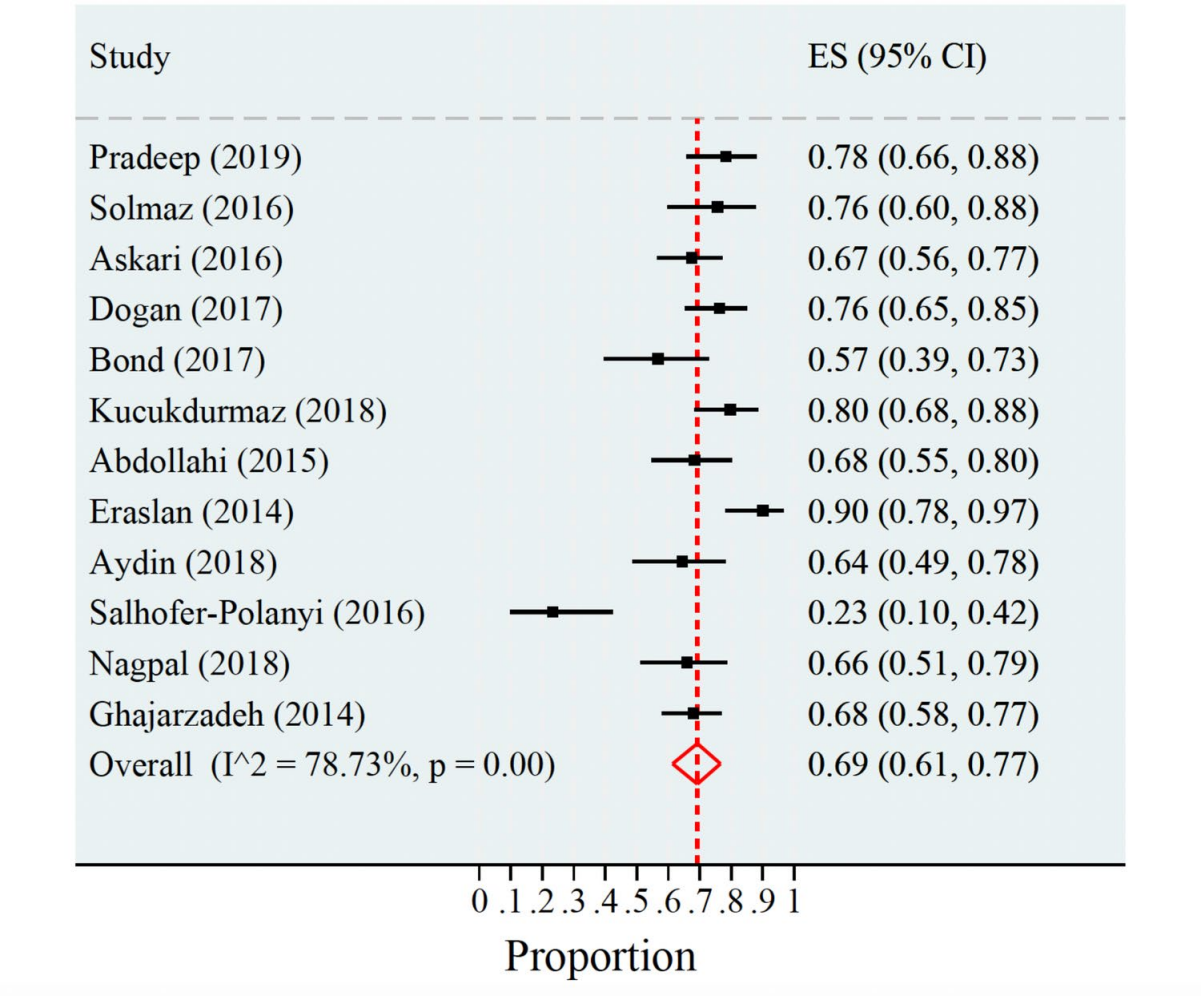
Pooled proportion with random-effects model of sexual dysfunction in migraine patients

A random-effects model was applied to the prevalence data to account for variation between studies. As demonstrated in Fig. 2, the estimated prevalence of sexual dysfunction in migraine patients pooled from the 12 studies that reported prevalence data was 69% (95% CI 61–77%). Between-study heterogeneity was substantial (I^2^ = 78.73%), indicating considerable variability in the true prevalence across studies. Sensitivity analyses were not performed.

### Quality assessment

The Joanna Briggs Institute (JBI) Critical Appraisal Checklist for Analytical Cross-Sectional Studies was used to determine the quality of studies used in this review as demonstrated in Table 4 (Aromataris et al. 2015). Eight studies were rated as “good” indicating a low risk of bias, seven were rated as “fair” indicating a moderate risk of bias, and one was rated as “poor” indicating a high risk of bias. Factors associated with study design and accounting for confounding variables accounted for the increased risk of bias.

**Table 4:** Assessment of bias within included studies via the JBI Critical Appraisal Checklist for Analytical Cross-Sectional Studies (Moola et al. 2015)

		JBI Checklist Criteria^a^								
Author	Year	1	2	3	4	5	6	7	8	Rating
Abdollahi	2015	-	-	✔	✔	/	/	✔	✔	Fair
Ahmed	2020	✔	✔	✔	✔	✔	?	✔	✔	Fair
Askari	2016	-	✔	✔	✔	✔	✔	✔	✔	Fair
Aydin	2018	-	-	?	?	✔	?	✔	✔	Poor
Bestepe	2011	✔	✔	✔	✔	✔	✔	✔	✔	Good
Bond	2017	✔	✔	✔	✔	✔	✔	✔	✔	Good
Dogan	2017	✔	✔	✔	✔	✔	✔	✔	✔	Good
Eraslan	2014	✔	✔	✔	✔	/	/	✔	✔	Good
Ertem	2020	✔	?	✔	✔	✔	?	✔	✔	Fair
Ghajarzadeh	2014	✔	✔	✔	✔	/	/	✔	✔	Good
Ifergane	2008	✔	-	✔	✔	✔	✔	✔	✔	Fair
Kucukdurmaz	2018	✔	✔	✔	✔	/	/	✔	✔	Good
Nagpal	2018	✔	✔	✔	✔	✔	✔	✔	✔	Good
Pradeep	2019	✔	✔	✔	✔	/	/	✔	✔	Good
Salhofer-Polanyi	2017	✔	✔	✔	✔	✔	?	✔	✔	Fair
Solmaz	2016	✔	✔	✔	✔	✔	?	✔	✔	Fair

^a^ Appraisal criteria questions are as follows:

1: Were the criteria for inclusion in the sample clearly defined?

2: Were the study subjects and the setting described in detail?

3: Was the exposure measured in a valid and reliable way?

4: Were objective, standard criteria used for measurement of the condition?

5: Were confounding factors identified?

6: Were strategies to deal with confounding factors stated?

7: Were the outcomes measured in a valid and reliable way?

8: Was appropriate statistical analysis used?

✔ = Yes

- = No

? = Unclear

/ = N/A

Rating scale

Good: all “Yes” or “N/A”

Fair: 1–2 "No" or "Unclear"

Poor: 3 + "No" or "Unclear"

## Discussion

### Prevalence

This review demonstrates that reported prevalence rates of sexual dysfunction in women with migraines can vary, ranging from 22.6% to 90%. If the review of prevalence rates is limited to studies determined to be “good” on quality assessment, then the prevalence range narrows to 56.8% to 90%. The overall wide range of prevalence indicates the presence of multiple outliers and an overall high burden of sexual dysfunction in this population. Variation was observed in prevalence rates between studies conducted in the same country. The two studies reporting significantly lower prevalence rates of 56.8% and 22.6% were conducted in the United States and Austria, respectively. Substantial heterogeneity was observed between studies, indicating that the true prevalences differ meaningfully across studies. These rates are consistent with previous research demonstrating high rates of sexual dysfunction in patients with chronic pain conditions (Flegge et al. 2023; Gruenwald et al. 2017). The development of sexual dysfunction in chronic pain is multifaceted (Flegge et al. 2023). Higher prevalence rates and poorer scores on measures of sexual dysfunction were demonstrated when considering the direct comparison of patients with migraines to matched controls, indicating a possible relationship between migraine and sexual dysfunction.

### Biological mechanisms

While the relationship between sexual dysfunction and migraine has been demonstrated in multiple studies, including this review, a clear pathophysiology has yet to be described (He et al. 2022; Torres-Ferrus et al. 2023). The mechanism of sexual dysfunction in the chronic pain population has been described as multifactorial and encompasses physical limitations, sleep disturbances, psychological factors, age, relationship satisfaction, and medication side effects (Flegge et al. 2023). Additionally, sexual dysfunction is associated with higher pain levels in patients with chronic pain conditions (Barr et al. 2024; Flegge et al. 2023). In this review, headache-related pain demonstrated a mixed association with sexual dysfunction and sexual distress. The lack of consensus regarding the relationship between migraine pain and sexual dysfunction aligns with the existing literature (He et al. 2022; Torres-Ferrus et al. 2023).

The role that other migraine characteristics, such as frequency and duration of headaches, may play in the development of sexual dysfunction is also complex. Increased migraine frequency and duration are associated with overall migraine-related disability and reduced quality of life (Pradeep et al. 2020; Shapiro et al. 2023). Importantly, increased headache frequency are also associated with increased rates of anxiety and depression amongst female migraine patients (Pradeep et al. 2020; Shapiro et al. 2023), which are factors that are strongly associated with the development of sexual dysfunction both in isolation and in the setting of chronic conditions (Basson and Gilks 2018; Torres-Ferrus et al. 2023). While previous research demonstrates an association between headache characteristics and factors contributing to sexual dysfunction, they do not explain the lack of consistency in the relationship between migraine characteristics and sexual dysfunction described in this review.

### Psychosocial mechanisms

Psychological factors play an important role in the development of sexual dysfunction, both in isolation and in the context of other chronic conditions. Migraines significantly increase the risk for developing depression (Amiri et al. 2019). The increased risk of depression is multifactorial and may be related to certain migraine characteristics, such as duration and frequency, biopsychosocial factors, brain development, genetics, stress, neurotransmitters, and hormonal factors (Pradeep et al. 2020; Zhang et al. 2019). Similarly, there is a strong relationship between the presence of migraines and the development of anxiety (Karimi et al. 2021).

This review demonstrates a positive correlation between depression and sexual dysfunction in most studies. Most notably, the sub-scores for desire, arousal, lubrication, orgasm, and satisfaction were impacted most frequently in this study, with some studies demonstrating a universal effect on all domains of sexual function. The prevalence of sexual dysfunction in female patients with depression is elevated at 83%, which is much higher than in the general population (Gonçalves et al. 2023). The effects of depression on the different domains of sexual function vary. A recent systematic review demonstrated a higher prevalence of sexual impairment in arousal at 47% and desire at 65% in women with depression and sexual dysfunction (Gonçalves et al. 2023). It is important to understand that the relationship between depression and sexual dysfunction is bidirectional, though the mechanism of this relationship is not yet well understood (Atlantis and Sullivan 2012). The use of antidepressants may further contribute to the development of sexual dysfunction, as this is a well-demonstrated side effect of many commonly used antidepressants (Atlantis and Sullivan 2012). The association between depression and sexual dysfunction is particularly important in the context of this review given the increased risk of depression in patients with migraines (Amiri et al. 2019). The association between migraines and depression, and subsequently sexual dysfunction, highlights the importance of treating comorbid psychological disorders in patients with migraines.

Most studies discussing the relationship between anxiety and sexual dysfunction in this review demonstrated a positive correlation. The study by Bond et al. demonstrated a 15% increase in the risk of sexual dysfunction amongst women with both migraine and anxiety. The findings of this review support the strong association between anxiety and sexual dysfunction in the existing literature (Basson and Gilks 2018). The prevalence of sexual dysfunction in patients with anxiety is also elevated, with rates between 33 and 75% (Herder et al. 2023). Anxiety primarily impacts the arousal and orgasm domains of sexual function through various mechanisms (Basson and Gilks 2018). The presence of anxiety demonstrated impacts on multiple domains of sexual function, including arousal and orgasm. The relationship between anxiety and arousal is complex and likely affected by both the physiological and cognitive aspects of sexual arousal (Bradford and Meston 2006). Anxiety may act as a distractor from sexual arousal via hypervigilance, obsessions, or worry. The increased sympathetic activity associated with sexual arousal may be misinterpreted as threatening by those with increased anxiety sensitivity secondary to state anxiety (Bradford and Meston 2006). There may be an interplay between anxiety, migraine, and sexual dysfunction, as one study demonstrated an increase in anxiety scores among female migraine patients with sexual dysfunction (Ahmed et al. 2021) in addition to multiple studies demonstrating increased rates of sexual dysfunction among female patients with migraine and anxiety.

### Management considerations

The likely multifactorial mechanism behind sexual dysfunciton in female migraine patinets underscores the importance of a multifaceted approach to treatment in this population. Importantly, these findings suggest a complex relationship between migraines, depression, anxiety, and sexual dysfunction. This highlights the importance of screening for sexual dysfunction in migraine patients with psychiatric conditions. The selection of antidepressant and antianxiolytic medications for the management of comorbid psychiatric conditions may be influenced by the presence of migraines. Additionally, thorough patient counselling should be employed when prescribing psychiatric medications with sexual side effects, as anti-depressant use has been associated with worsened sexual function in chronic pain patients (Barr et al. 2024).

### Limitations

There are some limitations in this systematic review to be addressed. The application of the findings of this review may be limited by the cross-sectional design of the included studies, which limits causal inference. The lack of an experimental study design limits the ability to draw directional conclusions. The variation in study quality, as determined by the Joanna Briggs Institute checklist, and the presence of heterogeneity between studies also limits the ability to compare findings across studies. Additionally, while most studies use the FSFI to measure sexual dysfunction, there was some variance in the screening tools used to measure this outcome, which may limit comparison between studies. The cutoff for defining sexual dysfunction with a given screening tool also varied, contributing to a lack of standardization across the studies. This is particularly relevant when discussing the impacts of migraine on various domains of sexual dysfunction, as the different screening tools used different domains. The screening tools used also did not take into account possible confounders, such as comorbid chronic illnesses and medication use, that could also influence sexual dysfunction.

It is also important to recognize that all the screening tools used in the included studies rely on participants to self-report their experiences, reducing the objectivity of the findings and making room for self-reporting bias. For example, the variations in the prevalence rates of sexual dysfunction demonstrated may suggest a variety in the subjective experiences of the participants included and interpretations of the questions in the reporting tools used to measure sexual dysfunction. These effects may be impacted by the effects of internalized stigmatization on the perception of sexual function, as this review included studies conducted across seven different countries. While cultural stigma and socioeconomic disparities broadly influence sexual and reproductive health, a recent systematic review demonstrated that religious and cultural values may limit discussions about sexuality in countries like Iran (Ouahid et al. 2025). Another Iranian study focused on the interplay of cultural, psychological, and systemic barriers that impede women from seeking help for sexual health concerns, impacting not only the extent of discussion on sexual health but also the prevalence of these concerns within particular communities (Taherpour et al. 2025). The demonstration of sexual dysfunction amongst women with migraine across numerous countries, including those in which stigmatization may have impacted reporting, indicates that these results are likely reproducible across the globe.

## Conclusion

Overall, this review demonstrates an increased prevalence of sexual dysfunction in female migraine patients. Clinicians should routinely assess the impact of migraine on sexual function. This review identifies factors associated with sexual dysfunction, including migraine characteristics like frequency and duration, pain associated with migraine, and psychological factors like anxiety and depression. Given the limitations of this review, future research may further characterize factors contributing to the relationship between sexual dysfunction and female patients with migraines.

## Supplementary Information

Below is the link to the electronic supplementary material.

Supplementary file1 (PDF 130 KB)

## Data Availability

All data analyzed in this systematic review are included in the submitted manuscript.
