# Supplementary material for: Sexual dysfunction in women with migraines: a systematic review
Source: Arch Womens Ment Health. 2026 Feb 3;29(1):31. doi: 10.1007/s00737-025-01650-6 (PMC12868073; doi:10.1007/s00737-025-01650-6)
Supplement: Supplementary file 1 — Supplementary file1 (PDF 130 KB) [file 737_2025_1650_MOESM1_ESM.pdf]

Title: Sexual Dysfunction in Women with Migraines: A Systematic Review
Journal Name: Archives of Women's Mental Health
Author Names: Karina Stech, BA, Anupama Jayachandran, BA, Huaqing Zhao, PhD, and Behnum Habibi, MD

Corresponding Author: Karina Stech
Email: karina.stech@gmail.com
Affiliation: Lewis Katz School of Medicine at Temple University, Philadelphia, PA, USA

**Supplemental Table 1**

| Study | Year | Assessment Tools | Sexual Function Outcome | Secondary Outcomes Contributing to Sexual Dysfunction |
|---|---|---|---|---|
| Abdollahi (Abdollahi et al. 2015) | 2015 | FSFI<br>VAS<br>HIT | High prevalence of sexual dysfunction (68.4%)<br>Desire (73.7%) and arousal (64.9%) most impacted domains of FSFI | Headache frequency positively correlated with sexual dysfunction**<br>No relationship between VAS and sexual dysfunction |
| Ahmed (Ahmed et al. 2020) | 2020 | ArFSFI<br>HDAS-A<br>HDAS-D<br>HIT-6TM<br>MSQLQ | Greater mean FSFI scores in migraine patients compared to healthy controls across all domains* | Female genital mutilation positively correlated with sexual dysfunction**<br>HADS-A positively correlated with sexual dysfunction*<br>HADS-D positively correlated with sexual dysfunction*<br>HITS-6TM positively correlated with sexual dysfunction*<br>VAS positively correlated with sexual dysfunction*<br>MSQLQ positively correlated with sexual dysfunction* |
| Askari (Askari et al. 2016) | 2016 | FSFI<br>BDI | High prevalence sexual dysfunction (67%) | Depression positively correlated with sexual dysfunction*<br>Positive correlation between migraine and depression*<br>Age positively correlated with sexual dysfunction* |
| Aydin (Aydin et al. 2018) | 2018 | FSFI | Sexual dysfunction in migraine (65%) > control (40%)*<br>Lower FSFI total and sub scores in migraine compared to control* | No significant difference in sexual dysfunction between migraine and TTH groups |
| Bestepe (Bestepe et al. 2011) | 2011 | ASEX<br>Socio-demographic questionnaire<br>VAS | Higher ASEX scores in migraine patients (14.95) compared to healthy controls (10.96)*<br>Lower number sexual activities in migraine patients compared to healthy controls*<br>Lower masturbation | No relationship between migraine characteristics and sexual dysfunction |

| | | | | |
|---|---|---|---|---|
| | | | frequency in migraine patients compared to healthy controls*<br>Decreased sex drive, sexual stimulation, vaginal lubrication, orgasmic satisfaction in migraine* | |
| Bond (Bond et al. 2017) | 2017 | FSFI<br>CES-D<br>GAD-7 | No difference in sexual dysfunction between patients (56.8%) and matched controls (54.1%)<br>FSFI scores between migraine and control groups were similar (24.5 ± 7.2 to 25.5 ± 4.6) | Anxiety positively correlated with sexual disability***<br>No correlation between migraine days, maximum pain intensity, or attack duration and sexual dysfunction |
| Dogan (Dogan et al. 2017) | 2017 | FSFI<br>BDI<br>BAI<br>MIDAS<br>VAS<br>Progesterone<br>Prolactin<br>FSH<br>LH | Sexual dysfunction in migraine (76.1%) > healthy control (37.1%)*<br>Mean migraine FSFI 22.9 ± 4.7 lower than control group (27.2 ± 4/7)*<br>Arousal, lubrication, orgasm, satisfaction, desire FSFI sub scores lower in migraine than control* | Depression positively correlated with sexual dysfunction**<br>MIDAS score negatively correlated with sexual satisfaction**<br>Prolactin positively correlated with desire**<br>Anxiety negatively correlated with arousal**<br>Depression negatively correlated with lubrication*<br>Anxiety negatively correlated with lubrication**<br>Prolactin positively correlated with lubrication***<br>Depression negatively correlated with orgasm**<br>FSH positively correlated with orgasm**<br>Headache attack frequency negatively correlated with sexual satisfaction** |
| Eraslan (Eraslan et al. 2014) | 2014 | FSFI<br>MIDAS<br>BDI<br>BAI | High prevalence of decreased sexual function (90%)<br>Mean FSFI score of 20.9 | No correlation between MIDAS score and sexual dysfunction<br>No correlation between migraine characteristics and sexual dysfunction<br>No correlation between BAI and sexual dysfunction<br>BDI positively correlated with sexual dysfunction*<br>BDI negatively correlated with desire**<br>BDI negatively correlated with arousal, lubrication, orgasm, and satisfaction* |
| Ertem (Ertem et al. 2020) | 2020 | GRISS<br>Headache characteristics | No difference in sexual dysfunction between patients and matched controls | NRS positively correlated with sexual dysfunction** |

| | | | | |
|---|---|---|---|---|
| | | NRS<br>BDI<br>BAI | | NRS positively correlated with anorgasmia** |
| Ghajarzadeh (Ghajarzadeh et al. 2014) | 2014 | FSFI<br>PSQI<br>BDI<br>VAS | High prevalence of sexual dysfunction (68%)<br>Mean FSFI score of (Bond et al. 2017).6 ± 8.8 | BDI negatively correlated with FSFI total score and desire, lubrication, pain*<br>BDI negatively correlated with arousal, orgasm, satisfaction**<br>PSQI negatively correlated with FSFI score**<br>Age negatively correlated with FSFI score*<br>No association between headache severity and sexual dysfunction |
| Ifergane (Ifergane et al. 2008) | 2008 | ISBI<br>MIDAS | Lower ISBI scores in migraine (49.5) vs control (68.9)**<br>Increased pain during intercourse in migraine patients**<br>Increased frequency of fear of sexual intercourse and penetration in migraine patients**<br>Decreased sexual satisfaction in migraine patients** | MIDAS negatively correlated with health domain of ISBI but not total ISBI score** |
| Kucukdurmaz (Kucukdurmaz et al. 2018) | 2018 | FSFI<br>MIDAS<br>FSDS-R<br>HDAS | High prevalence of sexual dysfunction (79.7%)<br>High prevalence of sexual distress in women with sexual dysfunction (67.2%)<br>Mean FSFI score of 19.25 ± 8.18 | Sexual distress positively correlated with anxiety*<br>Sexual distress positively correlated with MIDAS score*<br>Sexual distress positively correlated with depression**<br>No relationship between MIDAS or VAS and sexual dysfunction |
| Nagpal (Nagpal et al. 2018) | 2018 | FSFI<br>MIDAS<br>VAS | Sexual dysfunction in headache (66.3%) > control (30%)<br>Lower frequency of sexual intercourse in headache groups than controls*<br>Lower FSFI total scores in migraine compared to control**<br>Lower FSFI subscales in headache compared to control | MIDAS score positively correlated with sexual dysfunction in migraine**<br>Migraine duration, attack frequency, migraine severity as measured by VAS correlated with sexual dysfunction in migraine**<br>VAS score positively correlated with sexual dysfunction in migraine**<br>Depression positively correlated with sexual dysfunction in headache* |
| Pradeep (Pradeep et al. 2019) | 2019 | FSFI<br>MIDAS | High prevalence sexual dysfunction (78.3%)<br>Mean FSFI score of 23.1 +±- 4.76 | Duration of acute headache episodes positively correlated with sexual dysfunction**<br>Duration of acute headache episodes negatively correlated with desire** |

| | | | | |
|---|---|---|---|---|
| | | | | Migraine Days per Month positively correlated with sexual dysfunction** |
| Salhofer-Polanyi (Salhofer-Polyani et al. 2016) | 2016 | MSISQ-19<br>SF-36<br>BDI-II | 22.6% of migraine patients had sexual dysfunction<br>57.1% of migraine patients with sexual dysfunction had primary sexual dysfunction<br>71.4% of migraine patients with sexual dysfunction had secondary sexual dysfunction<br>71.4% of migraine patients with sexual dysfunction had tertiary sexual dysfunction<br>71.5% of migraine patients with sexual dysfunction had combinations of primary, secondary, and tertiary sexual dysfunction | Depression associated with sexual dysfunction**<br>Depression more prevalent in migraine patients (90.9%) compared to controls (34%)* |
| Solmaz (Solmaz et al. 2016) | 2016 | FSFI<br>Migraine Frequency | Sexual dysfunction in migraine (75.6%) > healthy control (7.3%)*<br>Mean migraine FSFI (21.59 ± 6.38) lower than control group (31.15 ± 3.60)*<br>Migraine FSFI subset scores lower in migraine vs control* | Migraine frequency positively correlated with sexual dysfunction*** |
| *P < 0.005<br>**P < 0.05<br>***P < 0.10 | | | | |

Supplemental Table 1: Study outcomes on sexual function in the setting of migraine and secondary outcomes relating to sexual dysfunction
